# The Care of Soldiers' Children

**Published:** 1919-10-18

**Authors:** 


### THE CARE OF SOLDIERS' CHILDREN.

PUBLIC attention is now being drawn to a scheme making special provision for the care of children of officers or men in the three Services who have died, or are on active service; children who, by reason of their mothers being dead, or from any other cause, are now suffering from the evils attendant upon neglect or want of proper care; and among the various post-war relief measures in which the nation, by popular sympathy and practical assistance, can show full appreciation of its indebtedness to the men who so magnificently defended the country and all its interests, none is more deserving of wholehearted support.

The official position stands as follows: By the War Pensions (Administrative Provisions) Act, 1918 (Section 9), it is imposed upon the Minister of Pensions to make special provision for these children. Already the Special Grants Committee, who have been entrusted with the duty by the Minister, are issuing new regulations which are intended to meet the case, and if their plans are forwarded by willing co-operation among the people there is every hope that much may be achieved towards attaining a very great and very necessary success.

The portion of the Committee's announcement with which those people now comfortably housed in the country are chiefly concerned relates to the conditions under which local committees are to place these unfortunate homeless children among the beneficial influences of life in private families, keeping them meanwhile under a necessary degree of observation. We feel, therefore, that the details of those official statements already issued should be brought home to every person so situated that they may be able to assist directly or indirectly in this movement, and we give below the main points of the latest issued announcements.

Each local committee, acting through a subcommittee—half of whose members are to be women and among them some with special knowledge of child care—will select suitable homes for the children, who will be placed in families belonging to their own religious denomination, while brothers and sisters will, as far as possible, be kept together. A weekly allowance of 12s. will be paid for a single child's maintenance, and where there is more than one child in a home 11s. for each of the others. With delicate children who require special attention, power is given to supplement these allowances. In addition, through the local war pensions committee, the Special Grants Committee may make allowance for outfit, boots, and clothing, travelling expenses, medical and dental treatment, surgical appliances, and higher education and training. Later, we read, it is the intention of the Minister of Pensions, as it must be the desire of the nation, that every child who, as the result of its father's sacrifice, is left without its natural home and guardians, shall be brought up in surroundings which are as like as possible to a real home, and shall be entitled to the full opportunities that care, education, and training can give, and to further the working of the scheme with all possible speed, it is urgently requested that any families who feel able to undertake this responsibility, will now communicate with their local war pensions committee, whose address can be readily obtained from the Post Office.

Such is the brief outline, and, although the extent to which this policy will become effective must largely depend upon the energy and zeal of the individual members of the local committees, yet the active sympathy of the general public remains of paramount importance to its successful working. It has been suggested, as a criticism, in fact, of the scheme, that owing to the grossly congested housing conditions now found in so many localities, it will be impossible to find accommodation in private houses for even a fraction of the number of these children, and accordingly the establishment of more orphanages has been recommended, but we are fully in accord with the official view that the method of private distribution should first be tried. The public has but to act in full and universal sympathy with this official endeavour and its success and the greater happiness of very many soldiers' children are assured. The possibilities of the voluntary scheme is instanced by the fact that in one of the most congested areas among the outlying London districts, the whole process of transferring 70 children from the care of the Poor-law Guardians and placing them with private families under supervision was completed within a few weeks. In the official note of this case it is stated that "private individuals gave to the Minister's agents that assistance which is another essential element for success."

